# Comprehensive analysis of AGPase genes uncovers their potential roles in starch biosynthesis in lotus seed

**DOI:** 10.1186/s12870-020-02666-z

**Published:** 2020-10-06

**Authors:** Heng Sun, Juanjuan Li, Heyun Song, Dong Yang, Xianbao Deng, Juan Liu, Yunmeng Wang, Junyu Ma, Yaqian Xiong, Yanling Liu, Mei Yang

**Affiliations:** 1 Key Laboratory of Plant Germplasm Enhancement and Specialty Agriculture, Wuhan Botanical Garden, Chinese Academy of Sciences, Wuhan, 430074 China; 2 Hubei Province Research Center of Engineering Technology for Utilization of Botanical Functional Ingredients, Hubei Key Laboratory of Quality Control of Characteristic Fruits and Vegetables, College of Life Science and Technology, Hubei Engineering University, Xiaogan, 432000 Hubei China; 3 University of Chinese Academy of Sciences, 19A Yuquanlu, Beijing, 100049 China; 4 Center of Economic Botany, Core Botanical Gardens, Chinese Academy of Sciences, Wuhan, 430074 China

**Keywords:** Lotus seed, Starch biosynthesis, Nutritional composition, AGPase gene, AGPase activity

## Abstract

**Background:**

Starch in the lotus seed contains a high proportion of amylose, which endows lotus seed a promising property in the development of hypoglycemic and low-glycemic index functional food. Currently, improving starch content is one of the major goals for seed-lotus breeding. ADP-glucose pyrophosphorylase (AGPase) plays an essential role in regulating starch biosynthesis in plants, but little is known about its characterization in lotus.

**Results:**

We describe the nutritional compositions of lotus seed among 30 varieties with starch as a major component. Comparative transcriptome analysis showed that AGPase genes were differentially expressed in two varieties (CA and JX) with significant different starch content. Seven putative AGPase genes were identified in the lotus genome (*Nelumbo nucifera* Gaertn.), which could be grouped into two subfamilies. Selective pressure analysis indicated that purifying selection acted as a vital force in the evolution of AGPase genes. Expression analysis revealed that lotus AGPase genes have varying expression patterns, with *NnAGPL2a* and *NnAGPS1a* as the most predominantly expressed, especially in seed and rhizome. *NnAGPL2a* and *NnAGPS1a* were co-expressed with a number of starch and sucrose metabolism pathway related genes, and their expressions were accompanied by increased AGPase activity and starch content in lotus seed.

**Conclusions:**

Seven AGPase genes were characterized in lotus, with *NnAGPL2a* and *NnAGPS1a*, as the key genes involved in starch biosynthesis in lotus seed. These results considerably extend our understanding on lotus AGPase genes and provide theoretical basis for breeding new lotus varieties with high-starch content.

## Background

*Nelumbo* is a unique genus of Nelumbonaceae, which comprises two extant species: *N. nucifera* Gaertn. widely distributed in Asia and northern Australia, and *N. lutea* Pers. which is distributed in America [1]. As a long-cultivated crop, lotus has versatile uses, parts of the plant have been used as a snack food, vegetable, medicine and ornamental horticulture. Based on distinct traits and agricultural uses, lotus cultivars can be classified into three groups: seed-lotus, rhizome-lotus and flower-lotus [2, 3]. Seed lotus can produce a higher yield of seed compared with the other two varieties. Currently, lotus seed products are very popular in daily diets in Asia, for example, the annual demand for lotus seed is about 50,000–75,000 tons in China. Mature lotus seed contains 40–60% of starch in the cotyledons, thus it is considered a rich source of starch, and is widely employed in the production of low-glycemic index food. In addition, lotus seed is rich in protein, vitamins, essential amino acids, and a variety of bioactive components with important nutritional and medicinal value.

As a non-structural carbohydrate, starch represents the most significant form of carbohydrate storage in plants. It is composed of two polymers of glucose, amylose and amylopectin, which have different molecular structures, the former is composed of unbranched chains of glucose monomers, while the latter is a branched polysaccharide [4]. Starch can be accumulated in photosynthetic and non-photosynthetic tissues through a highly complex processes [5]. The biosynthesis of starch begins in leaves and then transported to other storage organs in the form of disaccharides for starch synthesis. Sucrose transported from leaves to storage organs is successively catalyzed by sucrose synthase (SuSy), UDPglucose pyrophosphorylase (UDPase) and phosphoglucomutase (PGM) to produce glucose-6-phosphate, which is then transported into amyloplast for starch synthesis, which is catalyzed by PGM, ADP-glucose pyrophosphorylase (AGPase), starch synthase (SS) and starch branching enzyme (SBE) [6]. Furthermore, other important enzymes such as AGPase [7, 8], SS [9–11] and SBE [12, 13] have been identified and successfully applied in molecular breeding. Currently, the mechanism of starch biosynthesis process has been clarified plant species, such as *Arabidopsis*, potato, rice and corn [5, 14–16]. However, little is still known about the mechanism of starch biosynthesis in lotus seed.

Previous studies have shown that starch biosynthesis process plays an important role in the development of lotus seed [2, 17, 18]. Starch is rapidly accumulated in cotyledons from 9 to 20 days after pollination (DAP) in lotus [2, 17]. Proteomic analysis showed that AGPase was highly abundant at 25 DAP, and starch phosphorylase (StarchP) was significantly accumulated in mature lotus seed [17]. Comparative transcriptome between JX (‘Jianxuan 17’, a seed-lotus variety) with high starch content and CA (‘China Antique’) with low starch content showed that some enzyme encoding genes involved in starch biosynthesis, such as AGPase, soluble starch synthase (SSS) and SBE were up-regulated during lotus seed development [2].

AGPase plays an essential role in regulating glycogen and starch biosynthesis in bacteria and plants, respectively [19]. AGPase (EC:2.7.7.27) catalyzes the initial and major limiting step in the starch biosynthetic pathway, converting glucose-1-posphate (Glc1P) and ATP to ADP-glucose and pyrophosphate (PPi) [20–22]. AGPase is a heterotetramer composed of two small/catalytic subunits and two large/modulatory subunits [8, 23]. There are two isoforms of AGPases, known as the cytosolic and plastidial isoforms, based on its cellular localization. In the grain endosperm of the grass family, ADP-glucose is formed in the cytosol by the cytosolic isoform, and imported into plastids by plastid envelope located ADP-glucose transporter for starch biosynthesis. In contrast, ADP-glucose in most dicotyledonous plants is formed exclusively in the plastids by plastidial AGPase isoform [24]. Currently, AGPase genes have been identified in many species, such as rice, maize, *Arabidopsis* and barley [14, 25, 26]. Previous studies have shown that AGPase mutants exhibited a reduction in starch content, for example, in a near-starchless*Arabidopsis* TL25 mutant with an *adg1* gene encoding small subunit structural gene of AGPase [22, 27]. This deficiency in starch biosynthesis was also observed in a barley mutant, Risø 16, which has a large deletion within the coding region of the small subunit of the cytosolic AGPase [27]. To date, little information is available on the role of AGPase genes in lotus starch biosynthesis.

With the development and improved potential commercial application of lotus seed starch, the work of breeding new lotus varieties with high-starch content is crucial. In this study, we measured the nutritional composition of lotus seed among 30 varieties, and quantified starch, soluble sugar, protein and polyphenol content. As a key rate-limiting enzyme in starch biosynthesis, AGPase encoding genes were systematically identified and characterized by phylogenetic, expression pattern and co-expression network analyses based on the completed lotus genome (*Nelumbo nucifera* Gaertn.) sequencing data. The dynamic change of starch content and AGPase activity in lotus seed was detected in our study. Furthermore, two promising genes for starch biosynthesis, *NnAGPL2a* and *NnAGPS1a*, were identified in lotus seed. This study establishes a foundation for the understanding of starch biosynthesis pathway in lotus, and offer theoretical basis for molecular breeding of new lotus varieties with high-starch content.

## Results

### The nutritional compositions of lotus seed

**Fig. 1:**
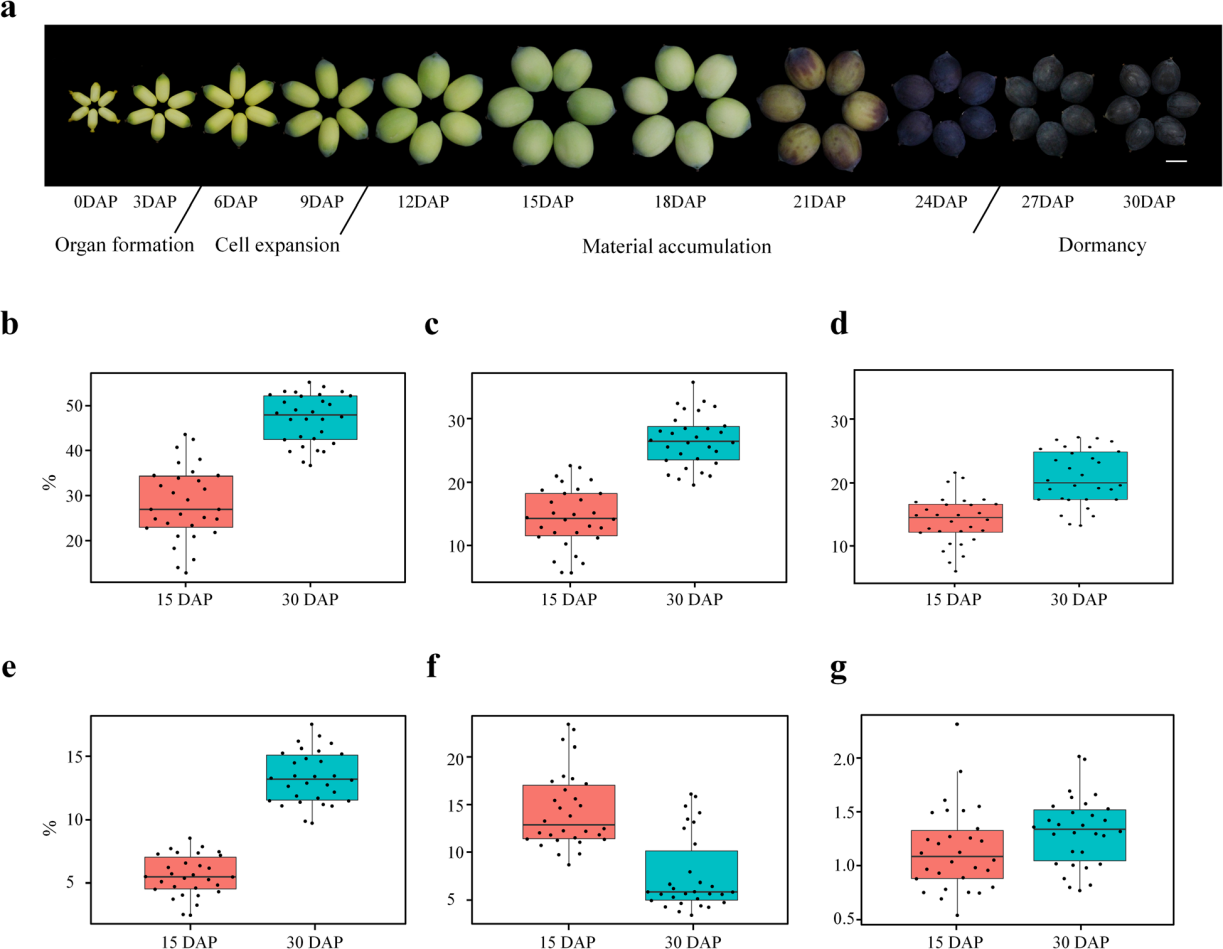
Morphological changes and nutritional compositions of lotus seed during development. **a** Morphological changes of lotus seed of JX (‘Jianxuan17’) during development. Bar = 1 cm. **b** - **g** The contents of starch **b**, amylose **c**, amylopectin **d**, protein **e**, soluble sugar **f** and polyphenols **g** in 15 DAP and 30 DAP in the seeds of 30 lotus cultivars

Lotus seed reaches mass maturity in about 30 days after pollination across four developmental stages, with organ formation at 1–3 DAP, cell expansion at 4–9 DAP, material accumulation at 10–25 DAP, and dormancy at 26–30 DAP. Every stage is accompanied by morphological changes, such as seed size and color (Fig. 1a). Seeds of 30 seed-lotus varieties were collected at 15 DAP and 30 DAP to determine the nutritional components, including total starch, amylose, amylopectin, protein, soluble sugar and polyphenol (Additional file 1: Table S1). For seeds collected at 15 DAP, total starch ranged from 12.79–43.60% with an average of 28.20%, amylose ranged from 5.67–22.58% with an average of 14.41%, amylopectin ranged from 5.68–21.33% with an average of 13.80%, protein ranged from 2.43–8.52% with an average of 5.55%, soluble sugar ranged from 8.66–23.43% with an average of 14.39%, and polyphenols ranged from 0.54–2.31% with an average of 1.15% (Fig. 1b - g). For seeds collected at 30 DAP, total starch ranged from 36.67–55.28% with an average of 47.12%, amylose ranged from 19.57–35.80% with an average of 26.58%, amylopectin ranged from 12.96–26.96% with an average of 20.54%, protein ranged from 9.7–17.53% with an average of 13.32%, soluble sugar ranged from 3.39–16.11% with an average of 7.56%, and polyphenols ranged from 0.77–2.01% with an average of 1.32% (Fig. 1b - g).

The contents of total starch, amylose, amylopectin and protein in 30 DAP lotus seeds were significantly higher than those in 15 DAP seeds (ANOVA, *P* ≤ 0.01), while the soluble sugar content in 15 DAP lotus seeds was significantly higher than that in 30 DAP lotus seeds (*P* ≤ 0.01), in contrast, no obvious change in the content of polyphenols was observed. In addition, there was no significant difference between amylose and amylopectin content in 15 DAP lotus seeds, while amylose content was significantly higher than the amylopectin content in 30 DAP lotus seeds (*P* ≤ 0.01). We found great differences in the nutritional composition among the 30 lotus varieties. For example, the coefficient of variation (CV%) for starch of seeds at 15 DAP, soluble sugar of seeds at 30 DAP and protein of seeds at 15 DAP was 29.10, 52.45 and 28.90, respectively (Additional file 1: Table S1).

### The AGPase genes are differentially expressed during lotus seed development

**Fig. 2:**
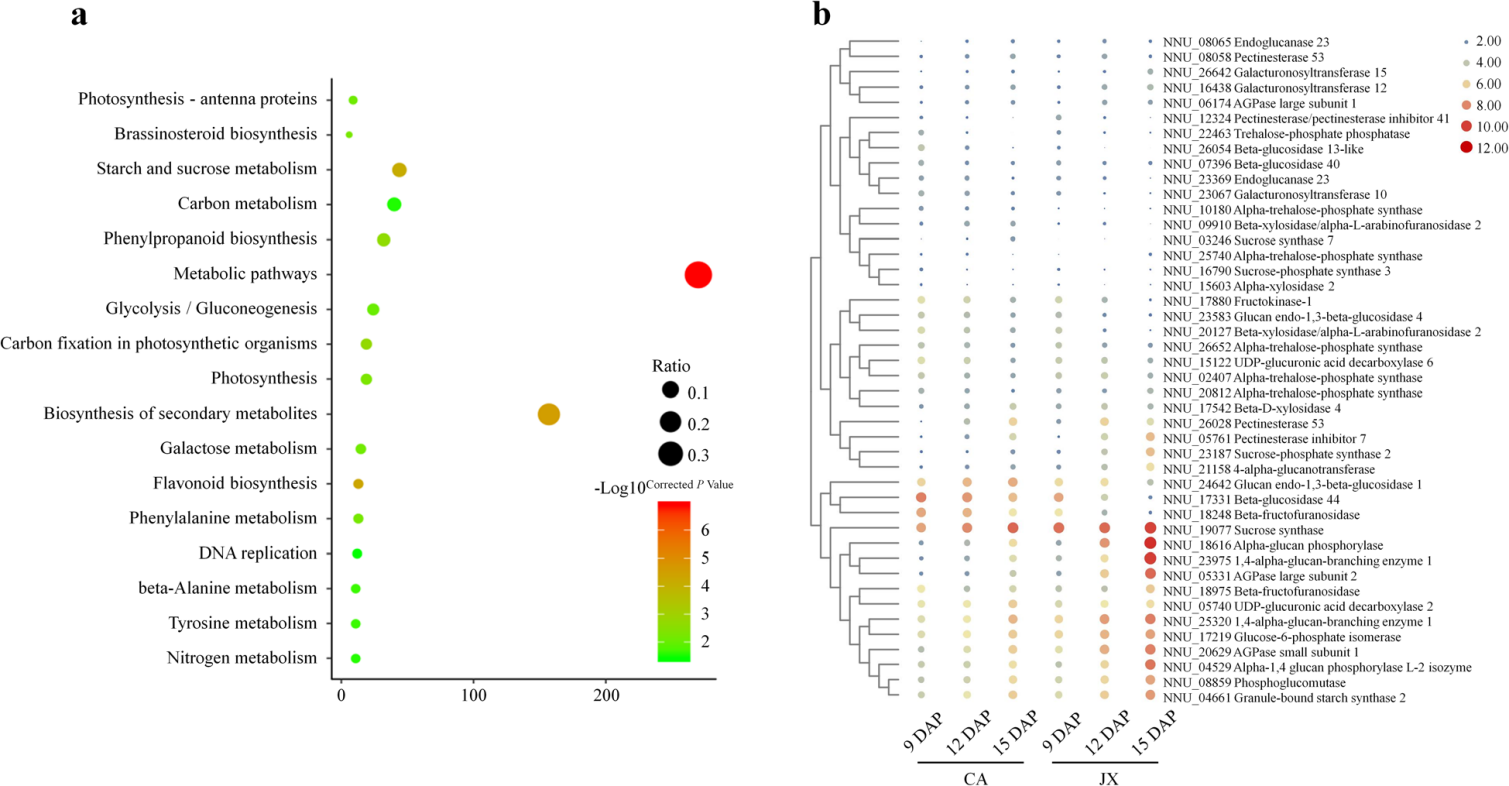
Functional analysis of differentially expressed genes (DEGs) during lotus seed development. **a** Functional enrichment analysis of 2895 common DEGs. **b** The expression of 44 DEGs involved in starch and sucrose metabolism

The comparative transcriptome analysis was performed to explore the differences in the molecular mechanism for seed development between CA and JX [2]. A total of 4416 and 6916 differentially expressed genes (DEGs) were identified in CA and JX from 9 DAP to 15 DAP, respectively, with 2895 common DEGs (Additional file 2: Table S2). KEGG analysis showed that these common DEGs were mainly involved in 17 pathways (corrected *P* value ≤0.05), including metabolic pathways, starch and sucrose metabolism and flavonoid biosynthesis (Fig. 2a). We found 44 genes involved in starch and sucrose metabolism pathway, and their expression patterns showed that 23 genes, including AGPase (NNU_05331, NNU_20629, NNU_06174), granule-bound starch synthase (NNU_04661) and 1,4-alpha-glucan-branching enzyme (NNU_25320, NNU_23975) were simultaneously up-regulated in CA and JX (Fig. 2b). Gene annotation showed that NNU_05331 and NNU_06174 encode the large subunit of AGPase, and NNU_20629 encode the small subunit. Interestingly, NNU_05331 and NNU_20629 were differentially expressed in CA and JX. The expression of NNU_05331 in JX was 16.90 and 27.13 fold higher than in CA, at 12 DAP and 15 DAP, respectively. Whilst, the expression of NNU_20629 in JX was 4.49 and 4.45 fold higher than in CA, at 12 DAP and 15 DAP, respectively. Previous study showed that JX could biosynthesize more starch than CA at 12 DAP and 15 DAP [2]. Therefore, it is tempting to speculate that the differences in AGPase expression could help explain the differences in starch accumulation in these two varieties.

### Identification of AGPase subunit genes in lotus genome

**Fig. 3:**
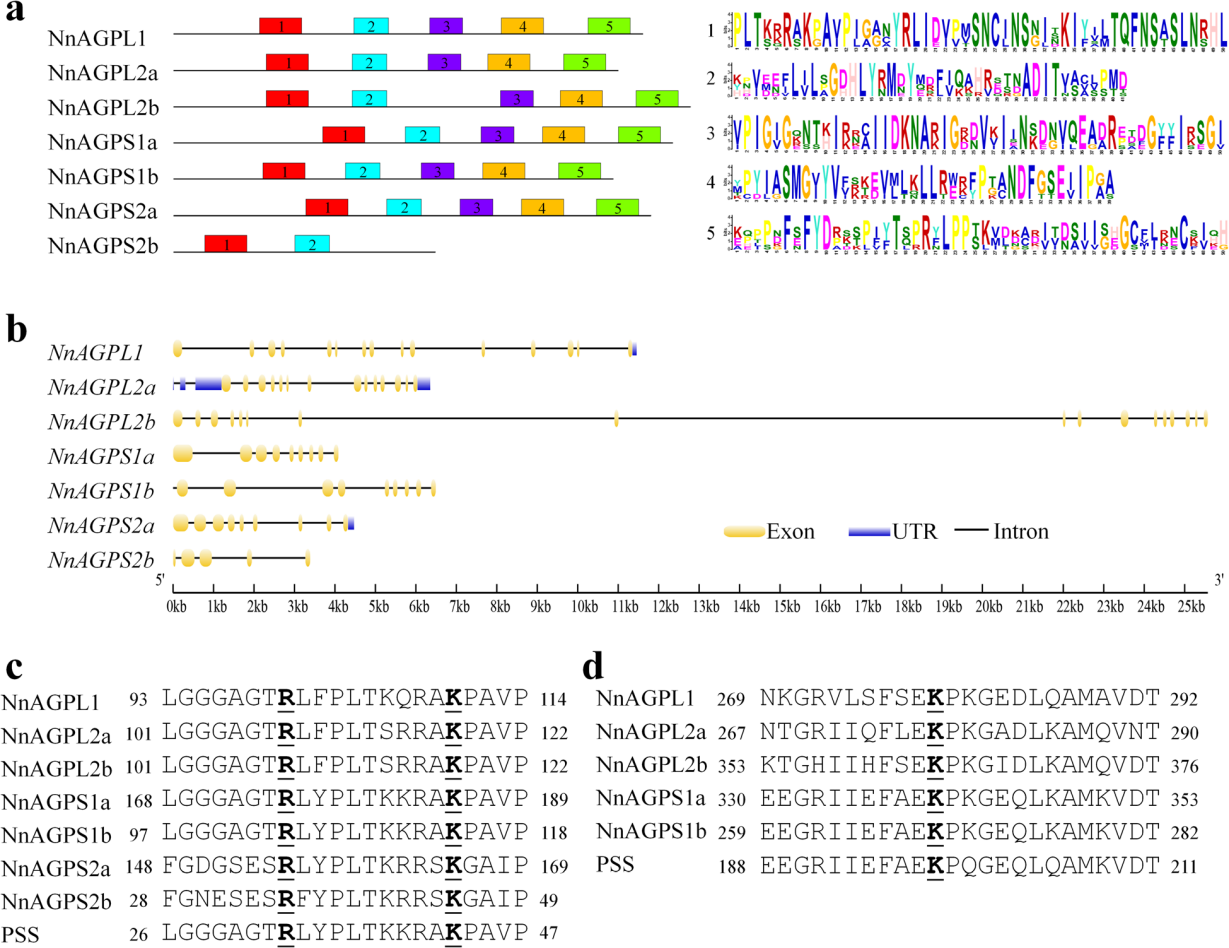
Sequence analysis of lotus AGPase genes. **a** Motif analysis of lotus AGPase genes. **b** The gene structure of lotus AGPase genes. **c** - **d** Sequence comparison of the catalytic site **c** and Glucose-1-phosphate binding region **d** of lotus AGPase genes. PSS: the potato small subunit gene, gene accession number is P23509

After a Blast search in the sacred lotus genome (*Nelumbo nucifera* Gaertn.), a total of seven AGPase genes were identified, including three *NnAGPL1*, *NnAGPL2a*, *NnAGPL2b* genes encoding the large subunit, and four *NnAGPS1a*, *NnAGPS1b*, *NnAGPS2a*, *NnAGPS2b* genes encoding the small subunit (Table 1; Additional file 3: Table S3). Four genes *NnAGPL1*, *NnAGPL2b*, *NnAGPS1a* and *NnAGPS2a* were distributed on Megascaffold_1, while *NnAGPL2a*, *NnAGPS2b* and *NnAGPS1b* were distributed on Megascaffold_6, Megascaffold_11 and Megascaffold_96, respectively. The full length of the AGPase subunit proteins ranged from 311 to 614 amino acids, while the molecular weights (Mw) of the large and small subunits ranged from 58.6 to 67.75 and from 35.21 to 65.2 kDa, respectively. The isoelectric points (pI) of the AGPase subunit proteins ranged from 6.18 to 9.01. Five conserved motifs were identified in six lotus AGPase genes, except for *NnAGPS2b* which only has two conserved motifs (Fig. 3a). Gene structure analysis showed that the large subunit contains 14 to 17 exons, while the small subunit contains 5 to 9 exons (Fig. 3b). In addition, sequence alignment of AGPase proteins against the PSS (the potato small subunit gene) revealed that the critical amino acids for catalysis (Arginine, **R** and Lycine, **K**) are in both AGPase subunit proteins. Five proteins including NnAGPL1, NnAGPL2a, NnAGPL2b, NnAGPS1a and NnAGPS1b had the same conserved binding site (Lycine, **K**) for Glc-1-P (Fig. 3c, d).

**Table 1:** Characteristics of lotus AGPase genes

Gene name	Gene ID	Loci	Length	Mw (kDa)	pI
*NnAGPL1*	NNU_06174	Megascaffold_1, 241,881,173–241,892,629	557	61.67	8.83
*NnAGPL2a*	NNU_05331	Megascaffold_6, 27,427,206–27,433,563	528	58.6	8.46
*NnAGPL2b*	NNU_17149	Megascaffold_1, 155,540,325–155,565,896	614	67.75	6.18
*NnAGPS1a*	NNU_20629	Megascaffold_1, 92,634,342–92,639,171	593	65.2	6.73
*NnAGPS1b*	NNU_21708	Megascaffold_96, 40,333–46,894	522	57.01	7.62
*NnAGPS2a*	NNU_10080	Megascaffold_1, 3,549,994–3,554,266	567	63.33	6.34
*NnAGPS2b*	NNU_11450	Megascaffold_11, 10,160,382–10,163,772	311	35.21	9.01

*Mw* molecular weight, *pI* Isoelectric points

### Evolutionary analysis of AGPase subunit genes

**Fig. 4:**
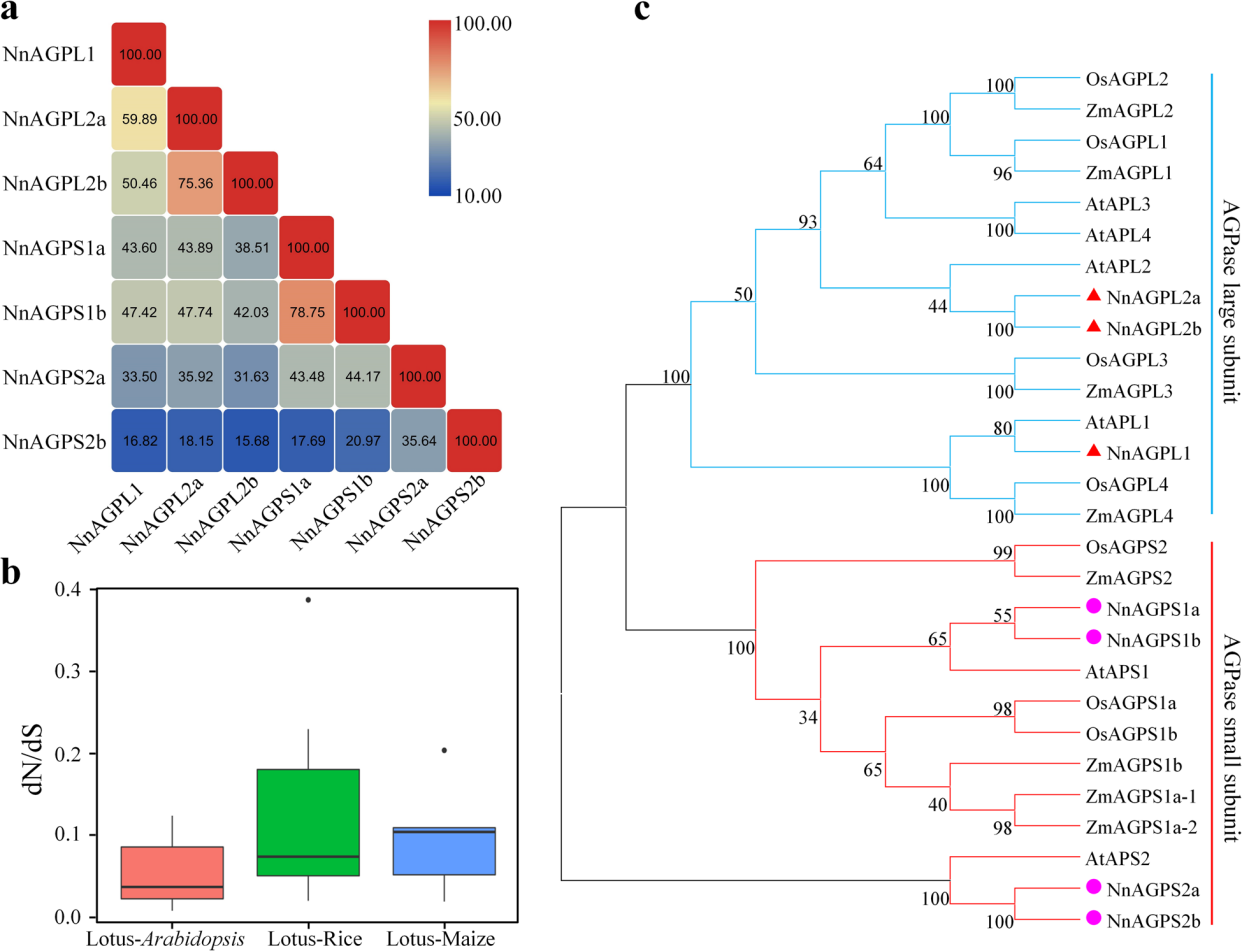
Evolutionary analysis of lotus AGPase genes. **a** Amino acid sequence homology of lotus AGPase proteins. **b** The ratio of non-synonymous to synonymous substitutions (dN/dS) between lotus AGPase genes and the homologous in *Arabidopsis*, rice and maize. **c** Phylogenetic tree of AGPase proteins

Protein sequence analysis showed that the amino acid homology of the large subunit genes was 50.46–75.36%, while for the small subunit genes was 17.69–78.75% (Fig. 4a), with NnAGPL2a-NnAGPL2b and NnAGPS1a-NnAGPS1b pairs showing the highest homology amino acid sequence for the large and small subunits, respectively. In contrast, lowest homology was observed between *NnAGPS2b* and any of the other six identified AGPase gene members. The non-synonymous (dN) and synonymous (dS) substitution rates of homologous gene pairs were calculated to explore evolutionary dynamics and selection pressures between lotus and other plants, which including *Arabidopsis*, rice and maize. All dN/dS < 1 was observed, indicating that purifying selection is acting on AGPase genes (Fig. 4b). A phylogenetic tree was constructed to gain insights into the evolutionary relationships of AGPase genes. All AGPase genes could be grouped into two subfamilies, and genes showed obvious differentiation between the large subunit and the small subunit (Fig. 4c). Compared to rice and maize, a closer evolutionary relationship of AGPase genes was detected between lotus and *Arabidopsis*.

### *NnAGPL2a* and *NnAGPS1a* are predominantly expressed in lotus

**Fig. 5:**
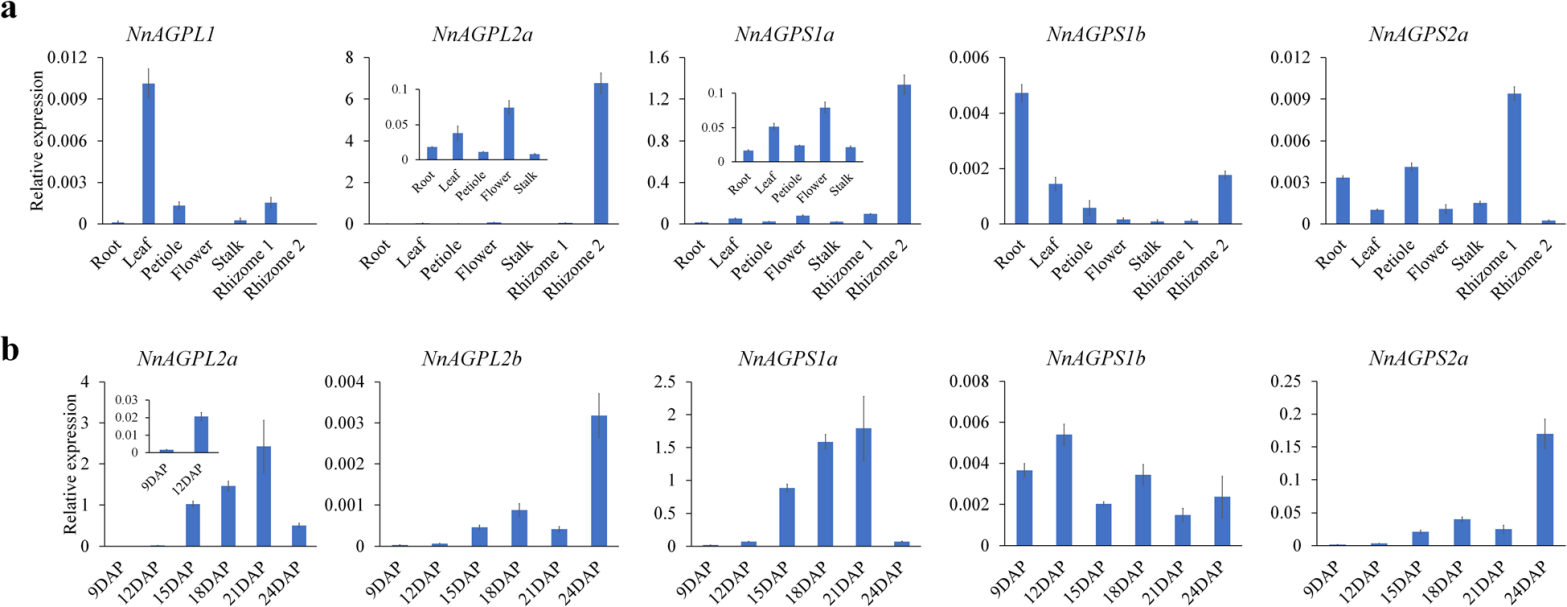
The tissue and spatio-temporal expression of lotus AGPase genes. **a** Expression analysis of the lotus AGPase genes in different tissues by qRT-PCR. **b** Expression analysis of the lotus AGPase genes during seed development by qRT-PCR. Bars represent means ± standard error (*n* = 3)

The expression patterns of lotus AGPase genes were investigated in different tissues of the cultivar JX, including root, leaf, petiole, flower, stalk, the stolon stage rhizome (rhizome 1) and the swelling stage rhizome (rhizome 2) by Real-time PCR. The expression of five genes were detected in at least one tissue except for *NnAGPL2b* and *NnAGPS2b* (Fig. 5a). *NnAGPL1* was highly expressed in leaf, and also found in petiole and rhizome 1, but not expressed in flower and rhizome 2. *NnAGPL2a*, *NnAGPS1a*, *NnAGPS1b* and *NnAGPS2a* were expressed in all tissues, with up-regulation of *NnAGPL2a* and *NnAGPS1a* in rhizome 2 than in rhizome 1. In addition, *NnAGPL2a* and *NnAGPS1a* showed overall higher expression abundances than other genes.

The expression patterns of lotus AGPase genes were also investigated during lotus seed development. For the large subunit genes, *NnAGPL2a* was continuously up-regulated from 9 DAP to 21 DAP, and *NnAGPL2b* showed an obvious expression change at 24 DAP (Fig. 5b). However, the expression abundances of *NnAGPL1* in developing seed was hardly detected. For the small subunit genes, three genes showed expression abundance except for *NnAGPS2b* (Fig. 5b). *NnAGPS1a* and *NnAGPS2a* were induced during seed development, however, *NnAGPS1b* showed no obvious change in trend. In addition, *NnAGPS1a* and *NnAGPS2a* showed similar expression patterns with *NnAGPL2a* and *NnAGPL2b*, respectively.

Based on the above results, we speculate that *NnAGPL2a* and *NnAGPS1a* are the predominantly expressed genes in AGPase large subunit and small subunit, respectively, especially in response to the development process in lotus seed and rhizome.

### Co-expression network analysis of *NnAGPL2a* and *NnAGPS1a*

**Fig. 6:**
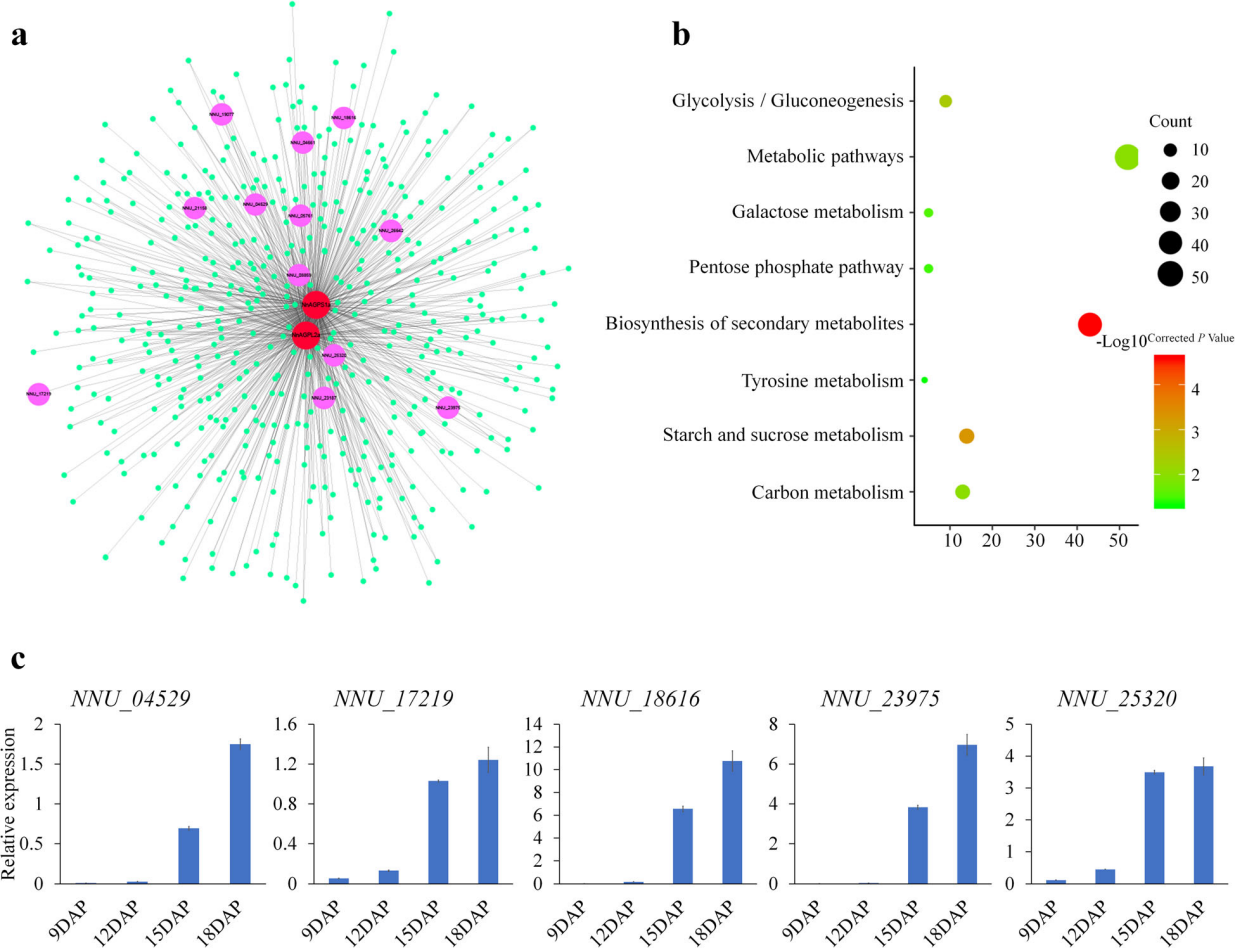
The co-expression network analysis of *NnAGPL2a* and *NnAGPS1a* in lotus seed. **a** Visualization of co-expression network of *NnAGPL2a* and *NnAGPS1a*. The magenta solid circles represent genes that are involved in starch and sucrose metabolism. **b** Functional enrichment analysis of the two *NnAGPL2a* and *NnAGPS1a* commonly co-expressed genes. **c** Expression analysis of five co-expressed genes by qRT-PCR. Bars represent means ± standard error (n = 3)

Co-expression network analysis is a powerful method for predicting gene function [28]. The predominantly expressed genes during lotus development, *NnAGPL2a* and *NnAGPS1a* were selected for this study. As a result, a total of 408 and 444 genes were co-expressed with *NnAGPL2a* and *NnAGPS1a*, respectively (|PCC| ≥ 0.9) (Fig. 6a; Additional file 4: Table S4), with 359 commonly co-expressed genes identified. KEGG analysis showed that eight pathways were enriched among these co-expressed genes (corrected *P* value ≤0.05), including the biosynthesis of secondary metabolites, starch and sucrose metabolism and carbon metabolism (Fig. 6b). Fourteen genes were involved in starch and sucrose metabolism, including granule-bound starch synthase (NNU_04661), alpha-1,4 glucan phosphorylase (NNU_04529) and 1,4-alpha-glucan-branching enzyme (NNU_23975, NNU_25320) (Additional file 5: Figure S1). Five genes were selected to verify the expression patterns by qRT-PCR, and all were significantly up-regulated from 9 DAP to 18 DAP and with similar expression pattern to *NnAGPL2a* and *NnAGPS1a* (Figs. 5b; 6c). Thus, it is likely that the role of *NnAGPL2a* and *NnAGPS1a* could be linked with these co-expressed genes to contribute to starch biosynthesis in lotus seed.

### Sequence variation of *NnAGPL2a* and *NnAGPS1a* between CA and JX

**Fig. 7:**
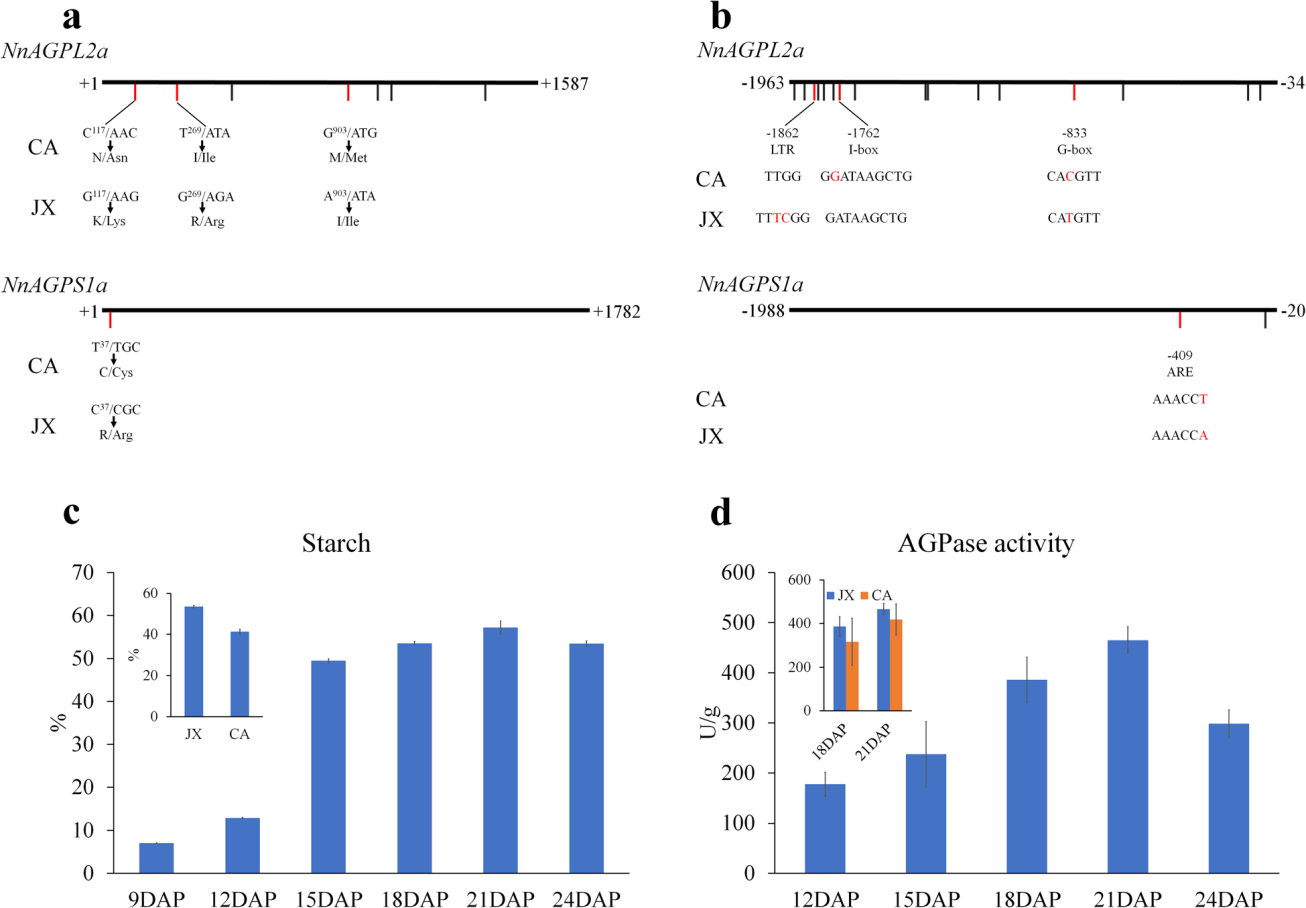
Sequence variation analysis and the dynamic changes in starch content and AGPase activity. **a** - **b**. Variation in the coding sequences of *NnAGPL2a* and *NnAGPS1a* **a** and their promoter regions **b** between CA and JX. **c** - **d**. The dynamic changes in starch content **c** and AGPase activity **d** during lotus seed development. Bars represent means ± standard error (n = 3)

Significant differences in seed size and starch content were detected between CA and JX varieties [2]. In order to investigate whether *NnAGPL2a* and *NnAGPS1a* have sequence variation in different lotus varieties, we cloned the coding sequences (CDS) and promoter regions of these two genes from CA and JX, respectively (Additional file 6: Figure S2; Additional file 7: Table S5). For *NnAGPL2a*, three nonsynonymous mutation and four synonymous mutation were identified in the CDS between CA and JX, the nucleotide polymorphism of C^117^/G^117^, T^269^/G^269^ and G^903^/A^903^ resulted in Asn^39^/Lys^39^, Ile^90^/Arg^90^ and Met^301^/Ile^301^, respectively (Fig. 7a; Additional file 6: Figure S2C). A total of 14 SNP and two Indel were identified in the promoter region of *NnAGPL2a*, and 12 variations were found to occur in the − 1000 to − 2000 bp region (Fig. 7b; Additional file 6: Figure S2d). Three variations caused changes in cis-elements, the SNP at position − 833 bp (C/T) in JX caused a change in G-box (CA**C**GTT→CA**T**GTT), the deletion at position − 1762 bp (G/−) in JX caused a change in I-box (G**G**ATAAGCTG→GATAAGCTG), the deletion at position − 1862 bp (TC/−−) in CA caused a change in LTR (TT**TC**GG→TTGG) (Fig. 7b).

Greater sequence conservation was detected in *NnAGPS1a* than in *NnAGPL2a* between CA and JX. A single SNP in the CDS and two in the promoter region were identified (Fig. 7; Additional file 6: Figure S2). The nucleotide polymorphism of T^37^/C^37^ in CDS resulted in Cys^13^/Arg^13^, while the SNP at position − 409 bp (A/T) in CA caused a change in ARE cis-element (AAACC**A**→AAACC**T**) (Fig. 7b).

### AGPase activity is increased during starch biosynthesis in lotus seed

Due to its potential role in regulating starch synthesis in plants, the dynamic change in starch content and AGPase activity in lotus seed of JX was detected in our study. The starch content and enzyme activity consistently increased from 9 DAP to 21 DAP (Fig. 7c, d). Starch synthesis peaked during 12DAP to 15DAP, then accumulation slowed until 21 DAP. JX had a higher starch content than CA, which is consistent with previous studies (Fig. 7c). Compared with starch biosynthesis, the enzyme activity showed a steady increasing trend until 21DAP, and then rapid decreased at 24 DAP (Fig. 7d). In addition, JX had a higher enzyme activity than CA at 18 DAP and 21 DAP in lotus seed (Fig. 7d).

## Discussion

Lotus seed is a product of sexual reproduction, and is largely consumed across Asia due to its rich in nutritional constituents. Previous studies on lotus seed has mainly focused on the processing technology, the structure and physicochemical properties of starch and the functional analysis of nutritional components, while the research progress on molecular mechanism of lotus seed development and quality traits has been slow [29–32]. In this study, we determined the content of key components in 30 seed-lotus varieties, these data will provide an important information on the scientific evaluation of lotus seed quality and the screening of germplasm resources for seed-lotus breeding.

Starch can be divided into three categories as low (< 20% amylose), medium (21–25%) and high (> 26%) based on amylose content [33]. Unlike most cereal crops, the starch in lotus seed belongs to natural high amylose, and some varieties contain more than 32% amylose, such as ‘Jianxuan 35’ (35.80%), ‘WBG_S1’ (32.78%) and ‘Honghua Jian Lian’ (32.44%). The starch in lotus seed can be easily retrograded to produce resistant starch, which has the potential to develop hypoglycemic functional food and low glycemic index food [34–36]. Our results indicate that the starch content in lotus seed still needs to be improved, with some main cultivars having their content lower than the average level of 47.12%, such as ‘Taikong 3’ (40.00%) and ‘Jingguang 1’ (42.66%). Previous studies have shown that understanding the mechanism of starch biosynthesis can successfully provide the molecular basis for breeding new varieties with high-starch content in crops, such as rice, corn and wheat [37–40]. Although the research on the mechanism of starch biosynthesis in lotus seed has attracted the attention of plant breeders, little is still known on the topic [2, 17, 18]. Previous studies have shown that starch accumulation is accompanied with a high mRNA and protein abundance of AGPase in lotus seed, indicating that AGPase plays an important role in this process [2, 17], thus warranting our study to analyze the mechanism of starch biosynthesis in lotus seed.

Here, we exploited the available genome data to systematically identify seven AGPase subunit genes in lotus. The number of AGPase genes in lotus was similar to those found in other plants, for example, seven in rice, eight in maize and six in *Arabidopsis* [14]. High variation was observed in small AGPase subunit genes than in large subunit genes, which is not consistent with previous reports that showed high amino acid sequence conservation of small subunit genes than large subunit genes [41]. The AGPase genes could be distinctly divided into two subfamilies in plants, indicating that large and small AGPase subunit genes have different evolutionary patterns (Fig. 4c). The lotus AGPase genes showed closer evolutionary relationship with genes in *Arabidopsis* than with rice and maize, thus supporting the conclusion that sacred lotus is a basal eudicot [1]. Selective pressure analysis showed purifying selection is acting as a primary force in the evolution of AGPase genes, which could suggest that the functions of AGPase homologous genes in different plant species are conserved.

The spatio-gene expression patterns can partly be used to predict gene function. In lotus, AGPase genes showed varied expression patterns in different tissues except for *NnAGPS2b*, suggesting that AGPase might play an important role in regulating the growth of lotus, especially in the starch synthesis tissues, such as seed and rhizome (Fig. 5). Two homologous gene pairs, *NnAGPL2a* - *NnAGPL2b* and *NnAGPS1a* - *NnAGPS1b*, showed an inconsistent expression patterns, which could suggest that functional differentiation of AGPase homologous genes has occurred in lotus. As a key enzyme in starch biosynthesis pathway, the regulatory properties of AGPase synergistically interact between the two subunits [7]. It is noteworthy that *NnAGPL2a* and *NnAGPS1a* are the predominantly expressed genes in lotus seed, suggesting these two are the key genes that might be involved in the regulation of starch biosynthesis and seed development. Differential expression of these two genes has been linked with difference in starch accumulation between CA and JX [2]. Phylogenetic analysis revealed that NnAGPS1a and NnAGPL2a are homologous to AtAPS1 and AtAPL2, respectively (Fig. 4c). AtAPS1 is the main small subunit in *Arabidopsis* predominantly regulating starch biosynthesis, with its mutant showing low starch phenotype, while AtAPL2 is the minor regulatory subunit in leaf with catalytic activity that could contribute to ADP-glucose synthesis in planta [7, 22]. We speculate that *NnAGPL2a* and *NnAGPS1a* are likely involved role in the regulation of starch biosynthesis in lotus seed, however, further studies to define the biological function and molecular mechanisms of these two genes are needed.

Domestication and breeding lead significant genetic changes in most crops, and identification of favored haplotypes can be used in molecular breeding [42]. In wheat, the haplotypes of two AGPase genes, *TaAGP-S1-7A* and *TaAGP-L-1B*, which are associated with thousand kernel weight (TKW), and the favored haplotypes underwent strong positive selection [37]. Here, we identified sequence variations in *NnAGPL2a* and *NnAGPS1a* between CA and JX. No variation was detected in the catalysis and Glc-1-P binding site, thus it would be worthy to determine whether variations in the CDS could affect the function of AGPase (Fig. 7a). The observed differential expression of *NnAGPL2a* and *NnAGPS1a* between CA and JX during lotus seed development, could be associated with the variation in the promoter region of these two genes (Fig. 7b). For example, the G-box mutation in *NnAGPL2a* promoter may affect the binding and regulatory activity of some environmental stress responsive transcription factors in JX, such as MYC and bZIP genes [43]. In addition, G-box is also closely associated with regulation of starch biosynthesis process, for example, a previous study showed that G-box plays a role in regulation of SBE and AGPase gene expression [44, 45]. Overall, these results partly reveal the genetic differentiation of *NnAGPL2a* and *NnAGPS1a*, and provide an important reference for identifying favored haplotype at the population level, which could be used in molecular breeding.

Rhizome is an important lotus storage organ, and starch is one of its most abundant components accounting for 10–20% fresh weight [46]. Previous studies have shown that AGPase gene is involved in the starch biosynthesis process in rhizome [3, 46]. Our data showed a higher AGPase enzyme activity at the initial swelling stage in comparison to stolon or late swelling stages in the rhizome of ZO (‘Zhou Ou’), which is a rhizome-lotus cultivar. Since *NnAGPL2a* and *NnAGPS1a* were the predominantly expressed and up-regulated genes during the rhizome swelling stage in two cultivars, JX and ZO (Fig. 5a; Additional file 8: Figure S3). We speculate that these two AGPase genes also regulate the starch biosynthesis in rhizome.

Our results showed that the expression patterns of *NnAGPL2a* and *NnAGPS1a* are accompanied with the changes in AGPase activity in lotus seed and rhizome (Figs. 5b; 7d; Additional file 8: Figure S3b). Therefore, enhancing the activity of AGPase by genetic engineering these two genes are likely to be a feasible way to increase the starch content in lotus seed and rhizome. Similar research strategies have been successfully applied in crops, such as rice, corn and wheat [47–50]. Transgenic rice plants expressing the potato AGPase large subunit *UpReg1* gene exhibited elevated photosynthetic capacity and starch levels in leaves, and increased seed biomass [47]. Overexpression of AGPase large subunit *TaAGPL1* significantly enhanced AGPase activity and the rate of starch accumulation in wheat grains [48]. Here, we provide important gene resources for future genetic improvement of starch accumulation in lotus varieties.

## Conclusions

**Fig. 8:**
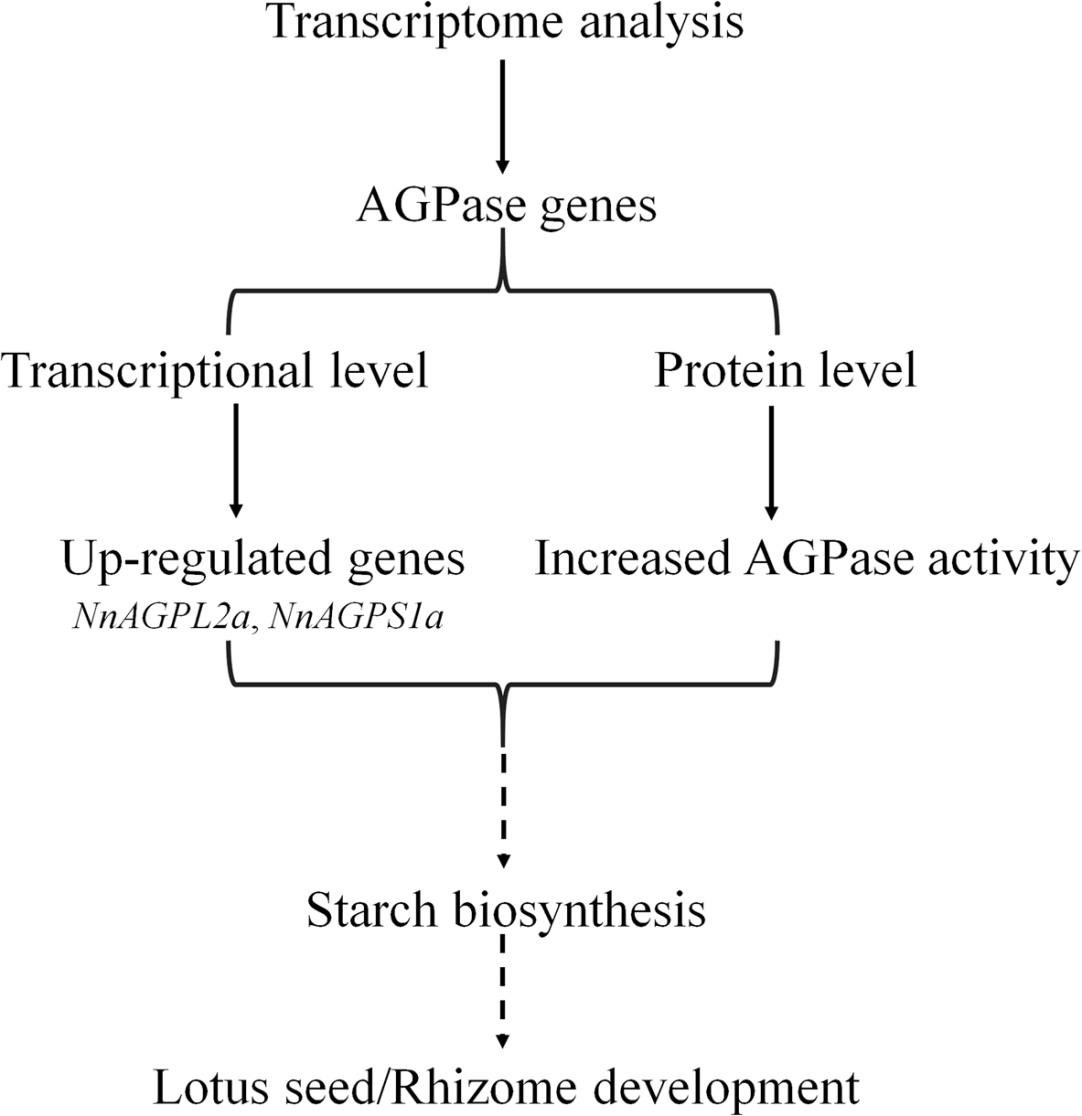
Summary model of this study. We identified AGPase genes involved in lotus seed development by comparative transcriptome analysis in lotus (*Nelumbo nucifera* Gaertn.). The expression pattern of *NnAGPL2a* and *NnAGPS1a* were accompanied by the increased AGPase activity and starch content

Improving the starch content is currently one of the major goals for seed-lotus breeding. In this study, we have revealed the nutritional composition including starch, soluble sugar, protein and polyphenols in the seed of 30 lotus varieties. These data provide important information on the scientific evaluation of lotus seed quality and for screening germplasm resources for seed-lotus breeding. Comparative transcriptome analysis showed that AGPase genes were differentially expressed in two varieties with significantly different starch content. Seven AGPase genes were characterized in lotus (*Nelumbo nucifera* Gaertn.), and their sequence conservation, evolution, expression pattern and co-expression network were analyzed. The expression patterns of *NnAGPL2a* and *NnAGPS1a* AGPase genes were accompanied by the increase in starch content and enhanced AGPase activity in lotus seed, thus are to be the key genes regulating starch biosynthesis in lotus seed (Fig. 8). This study presents a starting point for functional evaluation of AGPase genes in starch biosynthesis in lotus, and provides theoretical basis for breeding new lotus varieties with high-starch content.

## Methods

### Transcriptome analysis of lotus seed

To investigate the regulatory mechanism of starch accumulation during lotus seed development, the public transcriptome database corresponding to expression abundances in two lotus varieties CA and JX at various stages (9 DAP, 12 DAP, 15 DAP) were obtained from NCBI (https://www.ncbi.nlm.nih.gov/sra?term=SRP127765). The identification of differentially expressed genes (DEGs) was performed as previously described [2]. Kyoto Encyclopedia of Genes and Genomes (KEGG) enrichment analysis was implemented by KOBAS 3.0 [51]. The heatmap analysis of gene expression was visualized using TBtools [52].

For gene co-expression analysis, pearson correlation coefficient (PCC) was calculated based on the public transcriptome database to measure the co-expression relationships between two AGPase genes (*NnAGPL2a* and *NnAGPS1a*) and other genes [28]. Genes with |PCC| ≥ 0.9 were used for constructing co-expression network, and the network was visualized by Cytoscape software (3.4.0).

### Identification of AGPase genes from the sacred lotus genome

To identify AGPase subunit genes from the lotus genome, we obtained all the predicted protein sequences from lotus genome (*Nelumbo nucifera* Gaertn.) using gene annotation gff3 file [1]. The fully characterized AGPase subunit protein sequences of *Arabidopsis*, rice and maize were download from Tair (https://www.arabidopsis.org/index.jsp) and NCBI website (https://www.ncbi.nlm.nih.gov/). The protein BLAST program was performed to identify the lotus AGPase subunit genes. After removing the redundant sequences, the NTP_transferase domains of each lotus AGPase subunit protein amino acid sequences were analyzed using SMART online program (http://smart.embl-heidelberg.de/smart/set_mode.cgi? NORMAL = 1). The predicted molecular weight (Mw) and isoelectric points (pI) of AGPase subunit proteins were calculated using the ExPASy portal (http://web.expasy.org/protparam/). The complete amino acid sequences were analyzed using MEME software (http://meme-suite.org/index.html) to discover the conserved motifs of AGPase genes. Multiple sequence alignments of gene were carried out using ClustalX (ver.1.83) software with default settings.

### Phylogenetic analysis of AGPase genes in lotus

AGPase subunit protein sequences from four plant species including lotus, *Arabidopsis*, rice and maize were used for the phylogenetic analysis. The tree was constructed using MEGA7 software with Neighbour-Joining method as previously described [53, 54]. The homologous pairs of AGPase subunit genes between lotus and the three species were identified using BLASTP program. The homologous gene pairs were subsequently used to calculate non-synonymous (dN) and synonymous (dS) substitution rates to explore the evolutionary dynamics of AGPase genes. The dN/dS was calculated by Maximum Likelihood (PAML) yn00 program with the GMYN method [55].

### RNA extraction and qRT-PCR analysis

The lotus cultivars were grown in the experimental field at Wuhan Botanical Garden (N30°30′, E114°31′), Wuhan, China. Tissues and seeds were collected at different development stages, immediately frozen in liquid nitrogen, and stored at − 80 °C for later use. Total RNA was extracted using the Plant Total RNA Isolation Kit (Beijing Zoman Biotechnology Co., Ltd., Beijing, China). High-quality RNAs were reverse transcribed to cDNA using TransScript One-Step gDNA Removal and cDNA Synthesis SuperMix (Lot#M31212, Beijing TransGen Biotech Co., Ltd., Beijing, China). The qRT-PCR experiments were performed using StepOnePlus Real-time PCR System (Applied Biosystems, USA), and the relative gene expression level was calculated and normalized using *NnACTIN* (Gene ID NNU_24864) as the internal standard. Gene-specific primers for qRT-PCR were designed according to the gene coding sequences using Primer Premier 5.0 software and synthesized commercially (TIANYI HUIYUAN, Wuhan, China). The primers used for qRT-PCR are listed in Additional file 9: Table S6.

### Cloning *NnAGPL2a* and *NnAGPS1a* genes and their promoter regions

Gene and promoter specific primers (Additional file 9: Table S6) of *NnAGPL2a* and *NnAGPS1a* were designed based on the published lotus genome (*Nelumbo nucifera* Gaertn.) sequence database [1]. The coding sequence (CDS) of *NnAGPL2a* and *NnAGPS1a* were amplified using cDNA from 18 DAP seed of CA and JX as the template, the promoter region of these two genes was amplified using genomic DNA from CA and JX. The corresponding PCR fragments were cloned into pDONR ZEO for sequencing (TIANYI HUIYUAN, Wuhan, China). Comparison of the CDS and promoter sequences from JX and CA clones was done by ClustalX (ver.1.83) software with default settings. The cis-elements were analyzed using PlantCARE program [56].

### Assay of AGPase activity

The ADP-Glucose Pyrophosphorylase activity was detected using ADPG Pyrophosphorylase (AGP) Assay Kit according to the manufacturer’s instructions (Cat#BC0430, Beijing Solarbio Science & Technology Co., Ltd., Beijing, China). Samples from seed tissue at different stages (12, 15, 18, 21 and 24 DAP) and rhizome were collected and immediately frozen in liquid nitrogen, and stored at − 80 °C until use. All AGPase activity assays were performed in triplicate with one of the replicates assayed using 0.1 g of fresh tissue for every stage. A specific enzyme unit was defined as the amount of the enzyme that catalyzes the conversion of one nmol of NADPH per minute, per gram of tissue under specified assay conditions. The absorption wavelength was set to 340 nm and detection performed with an Infinite M200 Luminometer (Tecan, Mannerdorf, Switzerland).

### Determination of nutritional components in lotus seed

Thirty seed-lotus cultivars were kept and grown in the experimental field at Wuhan Botanical Garden (Wuhan, China) to analyze the nutritional composition of the seeds. Seeds were collected at 15 DAP and 30 DAP, and the collection of the materials complied with local and national guidelines. Subsequently, the germ was removed and the seeds were dried in an oven (DHG-9146A, Shanghai) at 100 °C for 1 h, and then dried at 65 °C to a constant weight. The dried seeds were ground into a powder and filtered by a 100-mesh sieve. The determination of nutritional components was performed at Wuhan ProNets Biotechnology Co,Ltd. (Wuhan, China). Three replicates were used for this assay.

Soluble sugars and starch content were measured using anthrone-sulfuric acid colorimetry method. Approximately 0.1 g of seed powder was mixed thoroughly with 10 ml of 80% ethanol in a centrifuge tube, boiled in in a water bath at 80 °C for 30 min and then centrifuged. The supernatant and pellet were used to determine the content of soluble sugar and starch content, respectively. A 2 ml volume of the supernatant was transferred to a new centrifuge tube, mixed with 0.5 ml anthrone-ethyl acetate (1 g anthrone dissolved in 50 ml of ethyl acetate) and 5 ml of concentrated sulfuric acid, then boiled in a water bath at 100 °C for 1 min, and cooled at room temperature. The absorbance was measured at 630 nm with a spectrophotometer (TU-1810D, Beijing, China). For starch content, the pellet was transferred into a 50 ml volumetric flask, mixed with 20 ml distilled water, and boiled in a water bath at 100 °C for 30 min. After boiling, 2 ml 9.2 mol/L perchloric acid were added. And extracted for 15 min, then cooled at room temperature. Finally, the tube was centrifuged and the supernatant was used to determine the content of starch by anthrone colorimetric assay.

The content of amylose was measured using iodine colorimetry [57]. Approximately 0.1 g of seed powder was mixed with 9 ml 1 mol/L NaOH solution in a centrifuge tube, boiled in a water bath at 100 °C for 10 min, after using distilled water for cooling capacity to 100 ml, 5 ml fluid was transferred to a new centrifuge tube, and 50 ml distilled water, 1 ml of 1 mol/L acetic acid and 1 ml iodine reagent (0.2 g iodine, 20 g potassium iodide dissolved in 100 ml distilled water) were added in turns. The absorbance was measured at 630 nm with a spectrophotometer.

Protein content was measured using coomassie brilliant blue method [58]. About 0.02–0.05 g seed powder was mixed with distilled water and ground to a homogenate, centrifuged at 4000 rpm/min for 10 min, then 1 ml of the supernatant was transferred to a new centrifuge tube, mixed with 5 ml of coomassie brilliant blue reagent (0.1 g coomassie bright blue dissolved in 50 ml 90% ethanol, added 100 ml 85% (W/V) phosphoric acid, and topped up to 1 L volume using distilled water). The absorbance was measured at 595 nm with a spectrophotometer.

Polyphenol content was measured using folin-ciocalteu colorimetry as previously described [59]. Approximately 0.02–0.05 g seed powder was mixed with 10 ml of 60% ethanol and hydrochloric acid (the final concentration is 0.024%) in a centrifuge tube, and boiled in a water bath at 75 °C for 50 min. The absorbance was measured at 765 nm with a spectrophotometer.

## Supplementary information

**Additional file 1: Table S1.** Summary statistics for nutritional components of lotus seed.

**Additional file 2: Table S2.** The expression of 2895 common DEGs during lotus seed development.

**Additional file 3: Table S3.** Protein sequences of lotus AGPase genes in lotus genome (*Nelumbo nucifera* Gaertn.).

**Additional file 4: Table S4.** The co-expressed genes of *NnAGPL2a* and *NnAGPS1a*.

**Additional file 5: Figure S1.** The expression of 14 co-expressed genes which were involved in starch and sucrose metabolism.

**Additional file 6: Figure S2.** Cloning of *NnAGPL2a* and *NnAGPS1a* genes and their promoter regions. a-b. The coding sequence (a) and promoter region (b) of *NnAGPL2a* and *NnAGPS1a* were amplified from CA and JX. c-d. Variation in the CDS of *NnAGPL2a* and *NnAGPS1a* (c) and promoter regions (d).

**Additional file 7: Table S5.** The CDS and promoter sequences of *NnAGPL2a* and *NnAGPS1a* in JX and CA.

**Additional file 8: Figure S3.** AGPase is involved in the development of rhizome. a. Illustrations of rhizome at different developmental stages. Bar = 1 cm. b. The activity of AGPase during rhizome development. Bars represent means ± standard error (*n* = 3). c. Expression analysis of *NnAGPL2a* and *NnAGPS1a* during rhizome development. Bars represent means ± standard error (n = 3).

**Additional file 9: Table S6.** Primer sequences used in this study.

**Additional file 10.** Original images for gels in this study.

## Data Availability

The main data supporting the results of this article are included within the article and the provided additional files. The genome data of *N. nucifera* ‘China Antique’ can be downloaded from GenBank (PID PRJNA168000, http://www.ncbi.nlm.nih.gov/Traces/wgs/?val=AQOG01). The public transcriptome data sets corresponding to expression abundances in two lotus varieties CA and JX are available in the Sequence Read Archive (SRA) of NCBI with accession number SRP127765.

## Abbreviations

AGPase: ADP-glucose pyrophosphorylase
CDS: Coding sequences
DEGs: Differentially expressed genes
dN: Non-synonymous
dS: Synonymous
Glc1P: Glucose-1-posphate
PGM: Phosphoglucomutase
pI: Isoelectric points
PPi: Pyrophosphate
SBE: Starch branching enzyme
SS: Starch synthase
SSS: Soluble starch synthase
SuSy: Sucrose synthase
UDPase: UDPglucose pyrophosphorylase
