# Supplementary figures and images for: Comprehensive analysis of AGPase genes uncovers their potential roles in starch biosynthesis in lotus seed
Source: BMC Plant Biol. 2020 Oct 6;20:457. doi: 10.1186/s12870-020-02666-z (PMC7541243; doi:10.1186/s12870-020-02666-z)

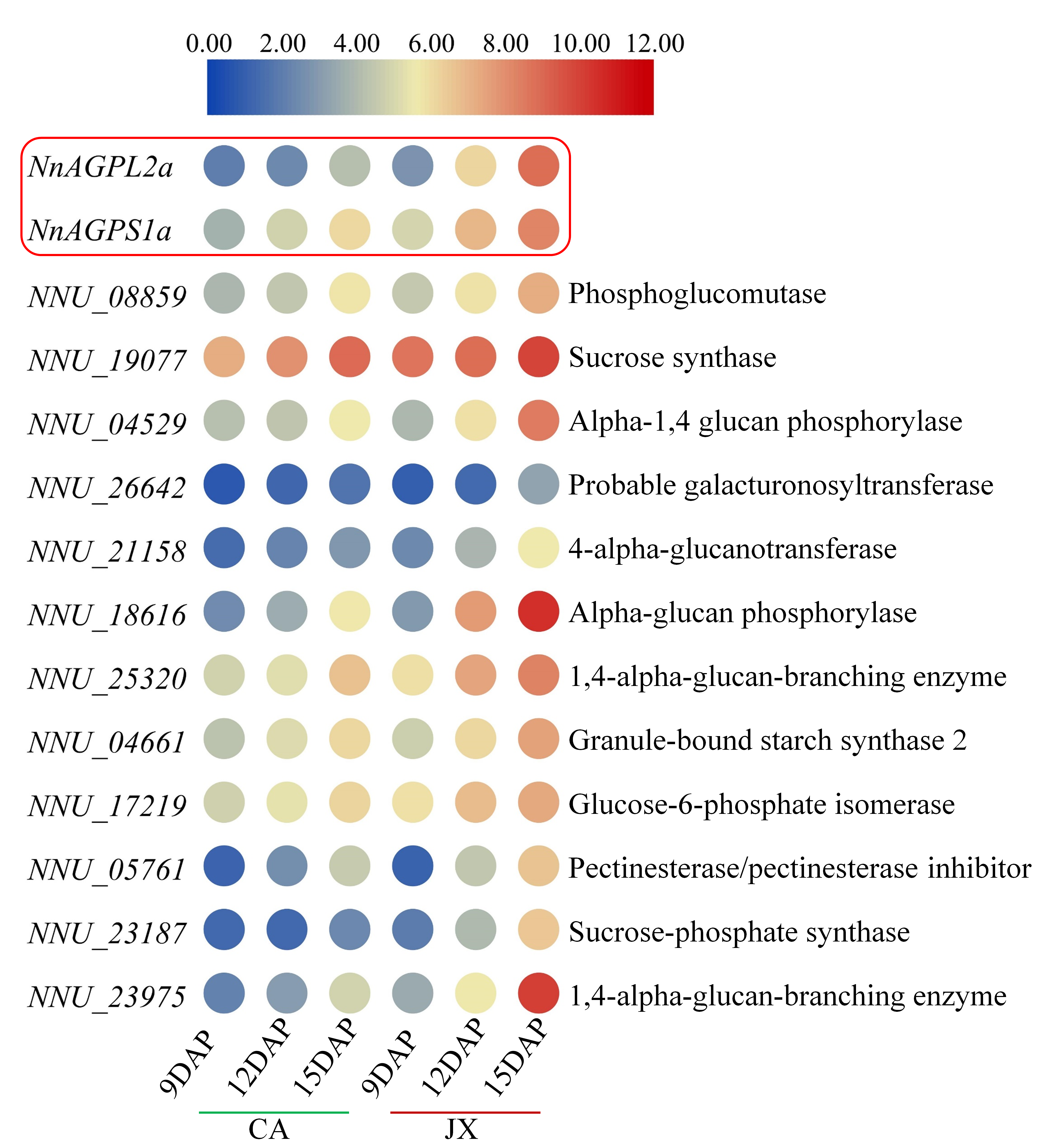

Supplement: Supplementary file 5 — Additional file 5: Figure S1. The expression of 14 co-expressed genes which were involved in starch and sucrose metabolism. [file 12870_2020_2666_MOESM5_ESM.tif]

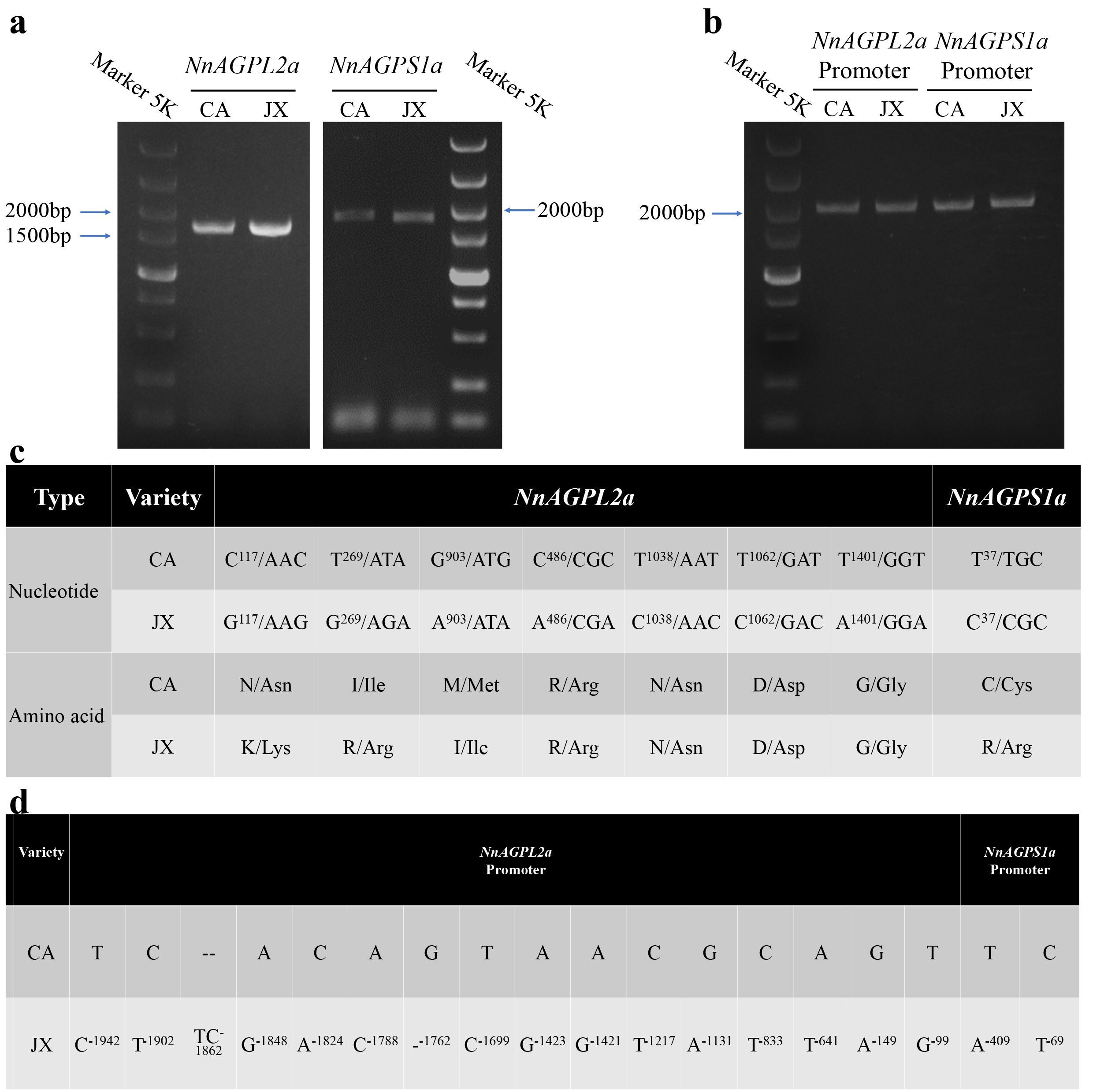

Supplement: Supplementary file 6 — Additional file 6: Figure S2. Cloning of NnAGPL2a and NnAGPS1a genes and their promoter regions. a-b. The coding sequence (a) and promoter region (b) of NnAGPL2a and NnAGPS1a were amplified from CA and JX. c-d. Variation in the CDS of NnAGPL2a and NnAGPS1a (c) and promoter regions (d). [file 12870_2020_2666_MOESM6_ESM.tif]

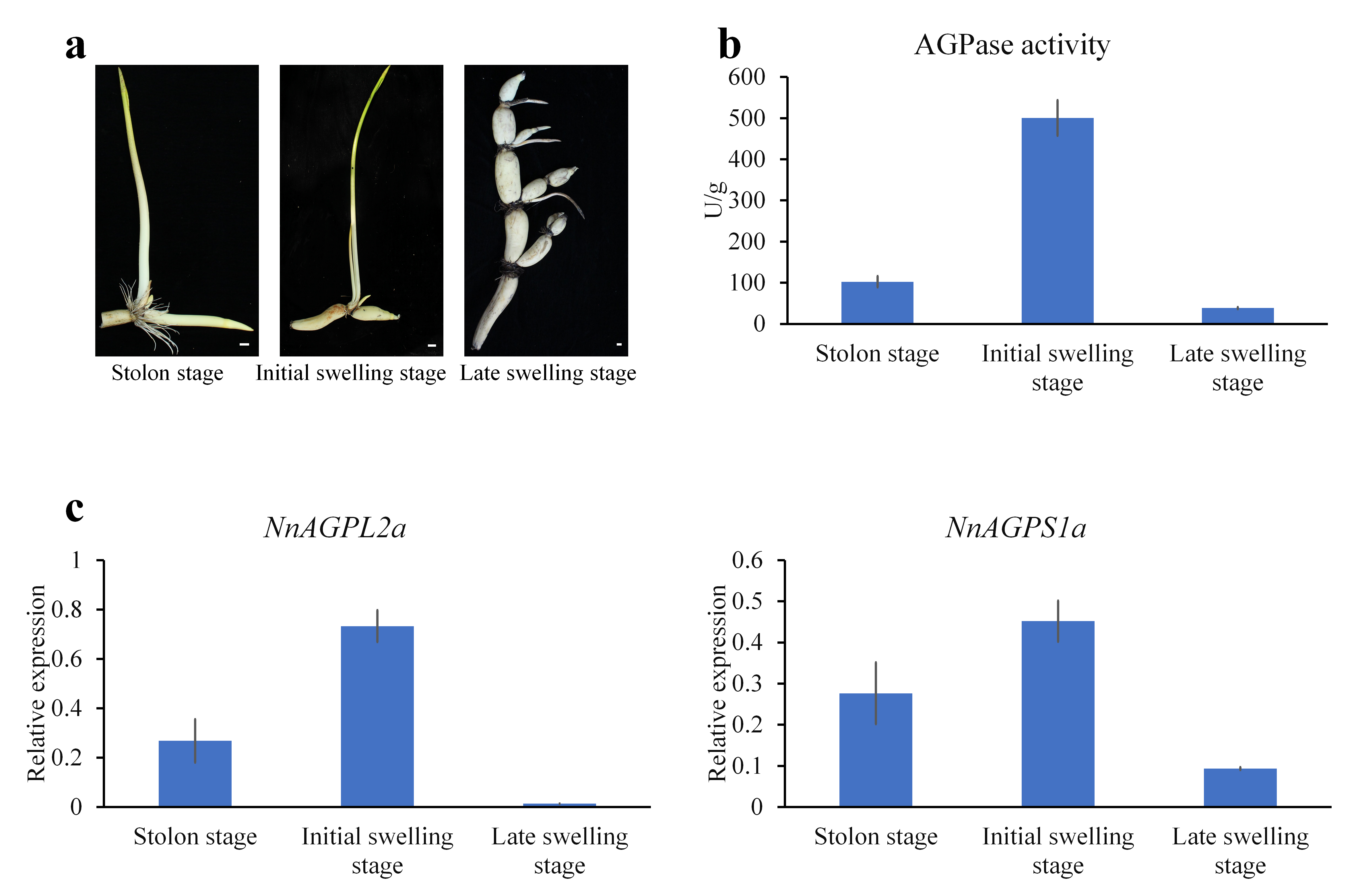

Supplement: Supplementary file 8 — Additional file 8: Figure S3. AGPase is involved in the development of rhizome. a. Illustrations of rhizome at different developmental stages. Bar = 1 cm. b. The activity of AGPase during rhizome development. Bars represent means ± standard error (n = 3). c. Expression analysis of NnAGPL2a and NnAGPS1a during rhizome development. Bars represent means ± standard error (n = 3). [file 12870_2020_2666_MOESM8_ESM.tif]

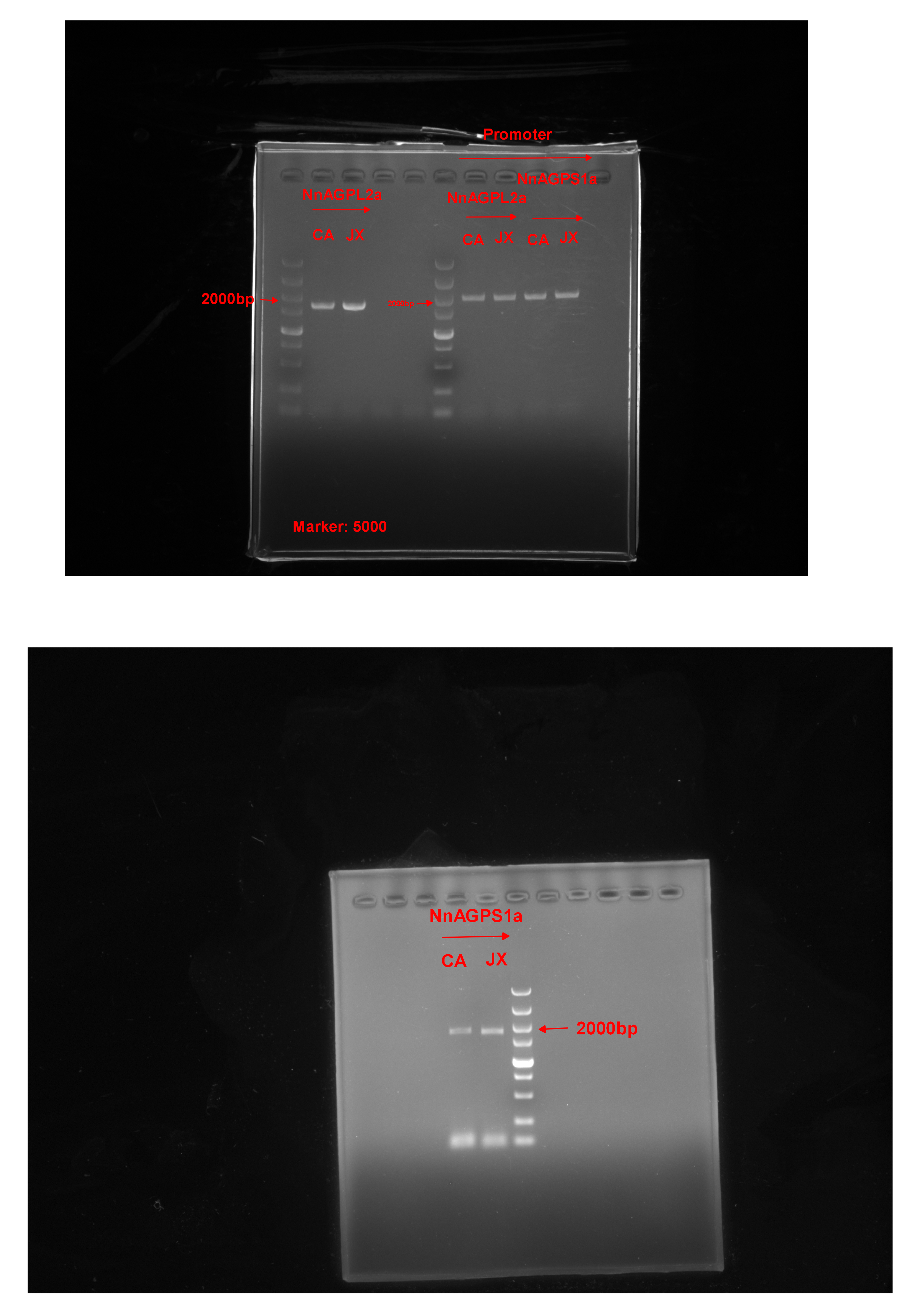

Supplement: Supplementary file 10 — Additional file 10. Original images for gels in this study. [file 12870_2020_2666_MOESM10_ESM.tif]
